# Comparison of the impact of allelic polymorphisms in *Pf*AMA1 on the induction of T Cell responses in high and low malaria endemic communities in Ghana

**DOI:** 10.1186/s12936-021-03900-1

**Published:** 2021-09-10

**Authors:** Ebenezer A. Ofori, John K. A. Tetteh, Augustina Frimpong, Harini Ganeshan, Maria Belmonte, Bjoern Peters, Eileen Villasante, Martha Sedegah, Michael F. Ofori, Kwadwo A. Kusi

**Affiliations:** 1 West Africa Centre for Cell Biology of Infectious Pathogens, Department of Biochemistry, Cell and Molecular Biology, College of Basic and Applied Sciences, University of Ghana, Legon, Accra, Ghana; 2 Department of Immunology, Noguchi Memorial Institute for Medical Research, College of Health Sciences, University of Ghana, Legon, Accra, Ghana; 3 Malaria Department, Naval Medical Research Center, Silver Spring, USA; 4 La Jolla Institute for Allergy and Immunology, La Jolla, CA USA; 5 Henry M. Jackson Foundation for the Advancement of Military Medicine, Bethesda, MD USA

**Keywords:** Malaria, T cells, IFN-γ ELISpot, Ghana, Apical membrane antigen 1 (*Pf*AMA1)

## Abstract

**Background:**

Malaria eradication requires a combined effort involving all available control tools, and these efforts would be complemented by an effective vaccine. The antigen targets of immune responses may show polymorphisms that can undermine their recognition by immune effectors and hence render vaccines based on antigens from a single parasite variant ineffective against other variants. This study compared the influence of allelic polymorphisms in *Plasmodium falciparum* apical membrane antigen 1 (*Pf*AMA1) peptide sequences from three strains of *P. falciparum* (3D7, 7G8 and FVO) on their function as immunodominant targets of T cell responses in high and low malaria transmission communities in Ghana.

**Methods:**

Peripheral blood mononuclear cells (PBMCs) from 10 subjects from a high transmission area (Obom) and 10 subjects from a low transmission area (Legon) were tested against 15 predicted CD8 + T cell minimal epitopes within the *Pf*AMA1 antigen of multiple parasite strains using IFN-γ ELISpot assay. The peptides were also tested in similar assays against CD8 + enriched PBMC fractions from the same subjects in an effort to characterize the responding T cell subsets.

**Results:**

In assays using unfractionated PBMCs, two subjects from the high transmission area, Obom, responded positively to four (26.7%) of the 15 tested peptides. None of the Legon subject PBMCs yielded positive peptide responses using unfractionated PBMCs. In assays with CD8 + enriched PBMCs, three subjects from Obom made positive recall responses to six (40%) of the 15 tested peptides, while only one subject from Legon made a positive recall response to a single peptide. Overall, 5 of the 20 study subjects who had positive peptide-specific IFN-γ recall responses were from the high transmission area, Obom. Furthermore, while subjects from Obom responded to peptides in *Pf*AMA1 from multiple parasite strains, one subject from Legon responded to a peptide from 3D7 strain only.

**Conclusions:**

The current data demonstrate the possibility of a real effect of *Pf*AMA1 polymorphisms on the induction of T cell responses in malaria exposed subjects, and this effect may be more pronounced in communities with higher parasite exposure.

**Supplementary Information:**

The online version contains supplementary material available at 10.1186/s12936-021-03900-1.

## Introduction

Malaria is a deadly vector-borne tropical disease of great public health concern. The disease is caused by the apicomplexan parasite *Plasmodium*, which is transmitted through the bite of an infected female *Anopheles* mosquito during a blood meal. With the increase in the number of existing tools recommended by the World Health Organization (WHO) for malaria control, malaria-related deaths have decreased over the last two decades in sub-Saharan Africa [1]. However, with these interventions in place, people, including children and adults, are still getting sick and dying from malaria. Hence, WHO has recommended the development of new interventions including vaccines to reinforce our hope of eradicating the disease [2]. RTS,S/AS01E (Mosquirix™) is the first and only malaria vaccine to date to demonstrate protection against malaria in children in phase 3 clinical trials [3]. However, its efficacy has been reported to wane with time [4] and this needs to be addressed in order to stay on track to meet the malaria eradication goal. Alternative vaccine design strategies are being explored for the development of more efficacious vaccines.

An effective malaria vaccine should be capable of inducing protective immune responses against variant forms of the parasite and in a genetically diverse population. *Plasmodium falciparum* apical membrane antigen 1 (*Pf*AMA1) has been investigated as a vaccine candidate in several clinical trials [5–8]. *Pf*AMA1 is found in the sporozoite, liver, and blood stages of the parasite [9] and therefore represents a multi-target antigen for vaccine development. It has been shown to be a target of both antibody [6, 10, 11] and T cell [12–16] responses. Antibodies to *Pf*AMA1 have been demonstrated to inhibit red blood cell invasion by merozoites [17].

In populations with high malaria endemicity, there is a measured high meiotic recombination rate and nucleotide substitutions in *P. falciparum* and these have been associated with increased drug and immune pressure on the parasites [18, 19]. For *Pf*AMA1, multiple nucleotide substitutions within the protein sequence result in the formation of allelic or polymorphic forms of the antigen in different parasite variants. As a direct result of these polymorphisms, antibodies to one allelic form of *Pf*AMA1 have been reported to bind less with other *Pf*AMA1 alleles because a substantial fraction of antibodies are towards strain-specific epitopes [17, 20]. While the effect of antigen polymorphisms has been clearly demonstrated for antibody responses, there is much less data on whether there is a similar effect of polymorphism on T cell recognition of epitopes within polymorphic antigens such as *Pf*AMA1. Sedegah et al. [21] have investigated the effect of single amino acid substitutions in immunodominant *Pf*AMA1 allelic epitopes on T cell response induction and report a significant effect of these single amino acid substitutions on TCR recognition of peptide-MHC complexes, and hence IFN-γ induction.

T cells are classically stimulated in the context of major histocompatibility complex (MHC) molecules and are known to bind to specific amino acid residues on the peptide sequence [22]. Furthermore, T cell receptors (TCRs) are typically known to interact with both the MHC molecules and the peptides being presented by the MHC molecules through specific residue identification [23–25]. MHC genes are amongst the most diverse human genes with thousands of variants within any population [26]. Hence, polymorphism within the peptides and/or within the MHC molecule will most likely affect MHC binding and TCR recognition [27]. This study therefore investigated the impact of allelic polymorphisms in selected *Pf*AMA1 peptide sequences from three strains of *P. falciparum* (3D7, 7G8 and FVO) on their function as targets of immunodominant T cell responses in high and low malaria transmission communities in southern Ghana.

## Methods

### Study communities and population

This cross-sectional study was conducted in two separate locations within the Greater Accra region of Ghana with different malaria transmission intensities; Obom is a high transmission community while Legon is a low malaria transmission community. Obom is in the Ga-South Municipality of Greater Accra Region of Ghana with a population of about 296,552 as projected in the 2018–2021 Revised Medium Term Development Plan [28]. Malaria transmission in Obom is perennial but the peak malaria season coincides with the main rainy season between June and August. The average yearly rainfall ranges between 790 to 1270 mm [29]. In 2018, malaria incidence by microscopy was 35.6% of all out-patient visits at the Obom Health Centre [30]. On the other hand, Legon is in the Ayawaso West municipality of the Greater Accra Region, with an estimated population of about 100,000 persons. In Legon, malaria transmission follows the rainfall pattern seen between May and June and the average annual rainfall is around 810 mm [31]. Malaria slide positivity is usually below 1% for most of the year [32]. The prevalence of *P. falciparum* is therefore higher in Obom compared to Legon [33].

### Subjects, sample collection and processing

Eligibility criteria for participation in the study were being between the ages of 18 and 50 years, not being pregnant or nursing for females and not being anaemic (Hb levels above 10 g/dl). Enrolled subjects had a normal medical history after screening. A total of 20 subjects, 10 from the high transmission area of Obom (s1 to s10) and another 10 from the low transmission area of Legon (s11 to s20) who met the inclusion criteria were recruited and screened for malaria parasites by rapid diagnostic test (RDT) kits and by light microscopy. Venous blood (60 ml) was obtained from each subject by venipuncture into heparinized tubes using aseptic techniques. Peripheral blood mononuclear cells (PBMCs) were isolated from blood by gradient centrifugation using Ficoll-Paque separating medium. After washing and counting, the cells were cryo-preserved at 20 million per milliliter in freezing mix (consisting of 90% FBS and 10% DMSO) at −80 °C overnight and transferred to liquid nitrogen until use for ELISpot assay. This study was conducted during the off peak/dry season (January, 2019), which has been reported to have significant reduction in parasite prevalence [29].

### Synthetic peptides selection

A set of 15 peptides from a previously generated set of predicted high-affinity MHC class I binders from the 3D7 parasite clone *Pf*AMA1 were selected for the present study. These selected peptides had some variant amino acid positions when compared to the corresponding *Pf*AMA1 sequences from the FVO and 7G8 parasite strains (Table 1). All peptides were synthesized commercially by Chiron Technologies (Clayton, Victoria, Australia). The 15 *Pf*AMA1 9-10mer peptides were grouped into six *Pf*AMA1 peptide sets, with each set containing the variant corresponding peptides from the three different parasite strains.

**Table 1:** *Pf*AMA1 peptide allele sets used for PBMC stimulation

Allelic set	Peptides	Sequence	Strain	HLA allele predicted for the 3D7 Pf variant peptide	HLA supertype	Amino acid residue positions
1	e1	AKD**KL**F**E**NY	FVO			278–286
	e2	AKD**K**SFQNY	7G8			278–286
	e3	AKDISFQNY	3D7	HLA B*15:03	B27	278–286
2	e22	DVY**H**PINEHR	7G8, FVO			36–45
	e23	DVYRPINEHR	3D7	A*68:01	A03	36–45
3	e24	EHREH**S**KEY	7G8			43–51
	e25	EHREHPKEY	3D7, FVO	HLA B*15:03	B27	43–51
4	e46	**D**FYK**N**N**E**YVK	7G8, FVO			200–209
	e48	HFYKDNKYVK	3D7	A*68:01	A03	200–209
5	e60	**KL**F**E**NYTYL	FVO			282–290
	e61	**K**SFQNYTYL	7G8			282–290
	e62	ISFQNYTYL	3D7	A*02:02 B*15:03 B*58:01	A02 B27 B58	282–290
6	e87	MTL**NG**MR**D**FY	FVO			193–202
	e88	MTLD**H**MR**D**FY	7G8			193–202
	e89	MTLDEMRHFY	3D7	A*30:02 A*29:02	A01 A01A24	193–202

Peptide sequences represent amino acid sequences deduced from variable regions of the *Pf*AMA1 3D7 (consensus), FVO and 7G8 strains. Peptide binding predictions were made for the 3D7 sequence using the NetMHC algorithm [34, 35] and based on recognition by the indicated specific HLA types. Predictions were based on IC50 values being less than 500 nM. Variable position(s) in corresponding peptide sequences of parasite variants are boldened and underlined in the sequences.

### Characterization of ELISpot IFN-γ-producing cells by T cell subset depletions

Subject-specific PBMCs were depleted of CD4 + T cells using anti-human CD4 + antibody-coated Dynabeads (Depletion MyOne™ SA Dynabeads® Invitrogen, USA) following the manufacturer's instructions. Flow cytometry was used to confirm the efficiency of CD4 + cell subset depletion.

### Ex vivo* ELISpot IFN-γ Assay*

IFN-γ ELISpot assay was performed as described earlier by [15, 36] using the unfractionated and CD8 + enriched PBMCs. Multiscreen plates (Millipore Corporation, USA) were coated with Monkey anti-human IFN-γ antibodies (Mabtech AB, USA) and incubated overnight at 4 °C. PBMCs (400,000 cells/well) from each subject were tested in duplicate with 10 μg/ml of each *Pf*AMA1 peptide. Concanavalin A (Con A, Sigma Aldrich, USA) at a concentration of 1.25 μg/ml was used as a cell viability positive control. Subject PBMCs that were incubated with medium only were used as negative controls. After PBMC incubation for 36 h, spots were detected by incubation with biotinylated anti-IFN-γ polyclonal antibody (Mabtech, USA) and subsequently with alkaline-phosphatase-conjugated streptavidin (Mabtech, USA). After plate development with chromogenic substrate for alkaline phosphatase (Bio-Rad, USA) and plate drying, the number of IFN-γ spots per well was counted using an automated Multispot plate reader (AID GmbH, Germany).

### Data analysis

The data acquired were exported to Microsoft Excel and IFN-γ activities of all peptides were calculated as the number of spot forming cells per million PBMCs (sfc/m). Response elicited by any peptide/stimulant was defined as positive based on our previously established thresholds [15, 36], which include (a) at least a doubling of sfc/m in test wells compared to negative control wells, and (b) a difference of at least 10 spots between test and negative control wells. Statistical analysis and graphics were done using Graph Pad prism (version 6.0, San Diego, CA, USA) and Microsoft Excel 2013. Flow cytometry analysis was done using FlowJo V10 software (Tree Star, San Carlos, CA, USA).

## Results

### Study participants

A total of 20 study subjects (10 from each of the two sites) who met the eligibility requirements and gave informed consent participated in the study. Study subjects were aged between 18 and 35 years, with an average age of 29 years at Obom and 26 years at Legon. All female subjects tested negative for pregnancy and all subjects were negative for malaria parasites by light microscopy and malaria RDT. For each subject, the ELISpot activity (sfc/m) for the unstimulated medium control was subtracted from the activities (sfc/m) for peptide or Con A control-stimulated cells prior to analysis. A summary of data for the six study subjects who made at least one positive peptide response is presented as Additional file 1.

### Ex vivo* ELISpot IFN-γ responses from unfractionated PBMCs to variant PfAMA1 peptides*

**Fig. 1:**
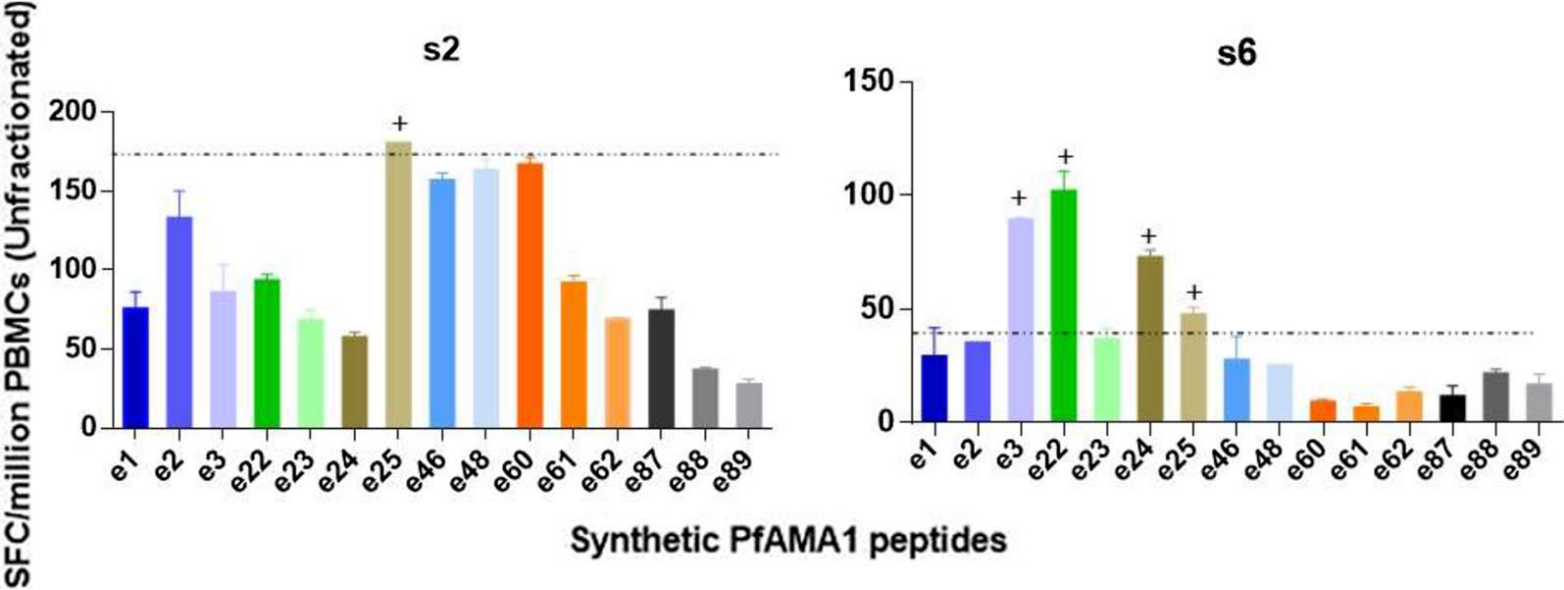
IFN-γ responses to variant *Pf*AMA1 synthetic peptides in subjects s2 and s6. Responses are against unfractionated PBMCs. Allelic peptides are presented as different shades of the same colour. Dotted horizontal lines indicate the positivity cut-off based on the positive response definition, and peptides labelled with + are those that had positive IFN-γ activity. SFC/million is Spot Forming Cells per million cells.

The magnitude of IFN-γ responses (sfc/m) from unfractionated PBMCs of two of the 20 study subjects following stimulation with 15 synthetic *Pf*AMA1 minimal epitopes is shown in Fig. 1. Two subjects (s2 and s6) from the high transmission area (Obom) made positive responses to four of the 15 peptides tested. The first subject (s2) responded positively to a single peptide e25, [EHREHPKEY, (3D7/FVO)], with no positive response to the corresponding allelic variant peptide e24 (see Table 1). The second subject (s6) responded to four of the 15 peptides tested (27%). The four peptides were e3, e22, e24, and e25 [AKDISFQNY (3D7), DVY**H**PINEHR (7G8/FVO), EHREH**S**KEY (7G8) and EHREHPKEY (3D7/FVO)], respectively. Two of these positive peptides (EHREH**S**KEY and EHREHPKEY) were variant peptides from different parasite strains. In the low transmission area, however, none of the subjects made positive responses to the 15 peptides tested based on the positivity criteria.

### *CD8* + *Enriched PBMCs IFN-γ activities against synthetic peptides*

**Fig. 2:**
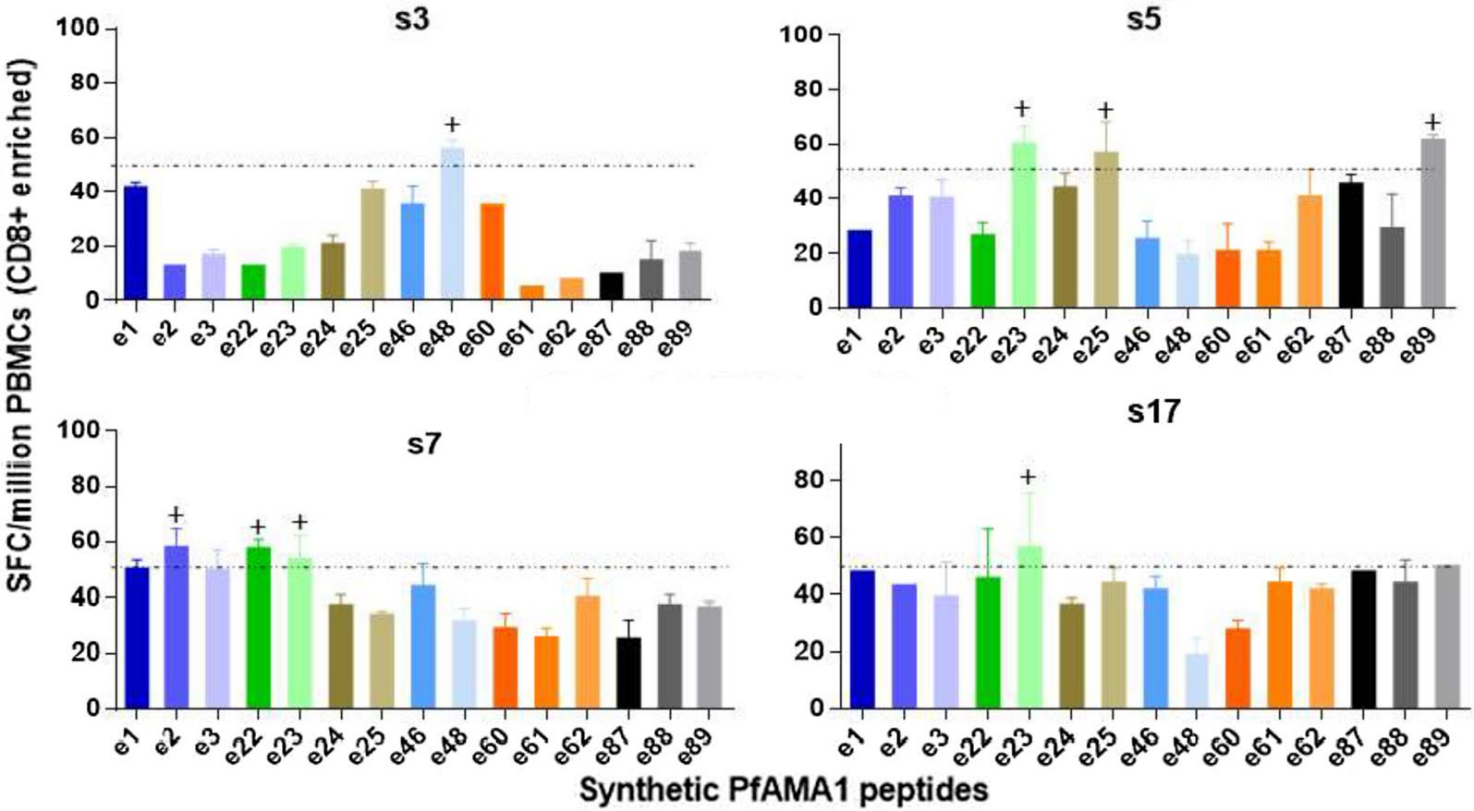
*Pf*AMA1 variant-specific IFN-γ responses of CD8 + enriched PBMCs from study subjects. Allelic peptides are presented as different shades of the same colour. Dotted horizontal lines indicate the positivity cut-off based on the positive response definition, and peptides labelled with + are those that had positive IFN-γ activity. SFC/million is Spot Forming Cells per million cells.

The CD4 + cell depletion protocol used achieved on average about 80% content of CD8 + T cells in fractionated PBMCs (Additional file 2). The magnitude of IFN-γ responses (sfc/m) from CD8 + enriched PBMCs against 15 synthetic *Pf*AMA1 peptides is shown in Fig. 2. CD8 + enriched PBMCs from three subjects from the high transmission area (Obom, s3, s5, s7) made seven positive responses to six peptides. Subjects s5 and s7 made positive responses to 3 peptides each, while subject s3 made a positive response to a single peptide [HFYKDNKYVK (3D7)]. However, in the low transmission area (Legon), CD8 + enriched PBMCs from subject s17 made a positive response to a single peptide [DVYRPINEHR (3D7)].

### Frequency of subjects’ responses to predicted PfAMA1 variant peptides in the two study areas

The frequency of variant peptide responses to study subjects are shown in Table 2. Generally, most of the peptides that recalled positive responses with subject PBMCs belonged to the 3D7 parasite clone. The five positive subjects from the high transmission area (Obom) and the single positive subject from the low transmission area (Legon) all responded to 3D7 peptides. Four of the five subjects from the high transmission area with positive 3D7 clone peptide responses also made positive responses to the variant peptides belonging to the other parasite strains, with s3 being the only subject that responded to a 3D7 peptide alone*.*

**Table 2:** Parasite variant-specific responses at the two study sites

Study site	Subject	PBMC fraction	Positive peptides	Parasite Strain
Obom (high transmission area)	s2	Unfractionated PBMCs	EHREHPKEY	3D7/FVO
	s3	CD8 + enriched PBMCs	HFYKDNKYVK	3D7
	s5	CD8 + enriched PBMCs	DVYRPINEHR EHREHPKEY MTLDEMRHFY	3D7 3D7/FVO 3D7
	s6	Unfractionated PBMCs	AKDISFQNY DVY**H**PINEHR EHREH**S**KEY EHREHPKEY	3D7 7G8/FVO 7G8 3D7/FVO
	s7	CD8 + enriched PBMCs	AKD**K**SFQNY DVY**H**PINEHR DVYRPINEHR	7G8 7G8/FVO 3D7
Legon (low transmission area)	S17	CD8 + enriched PBMCs	DVYRPINEHR	3D7

The underlined amino acids are the polymorphic residues relative to the 3D7 variant. All sequences in this table gave positive IFN-**γ** responses against the indicated PBMC fractions

### Positions and frequency of amino acids substitutions

**Fig. 3:**
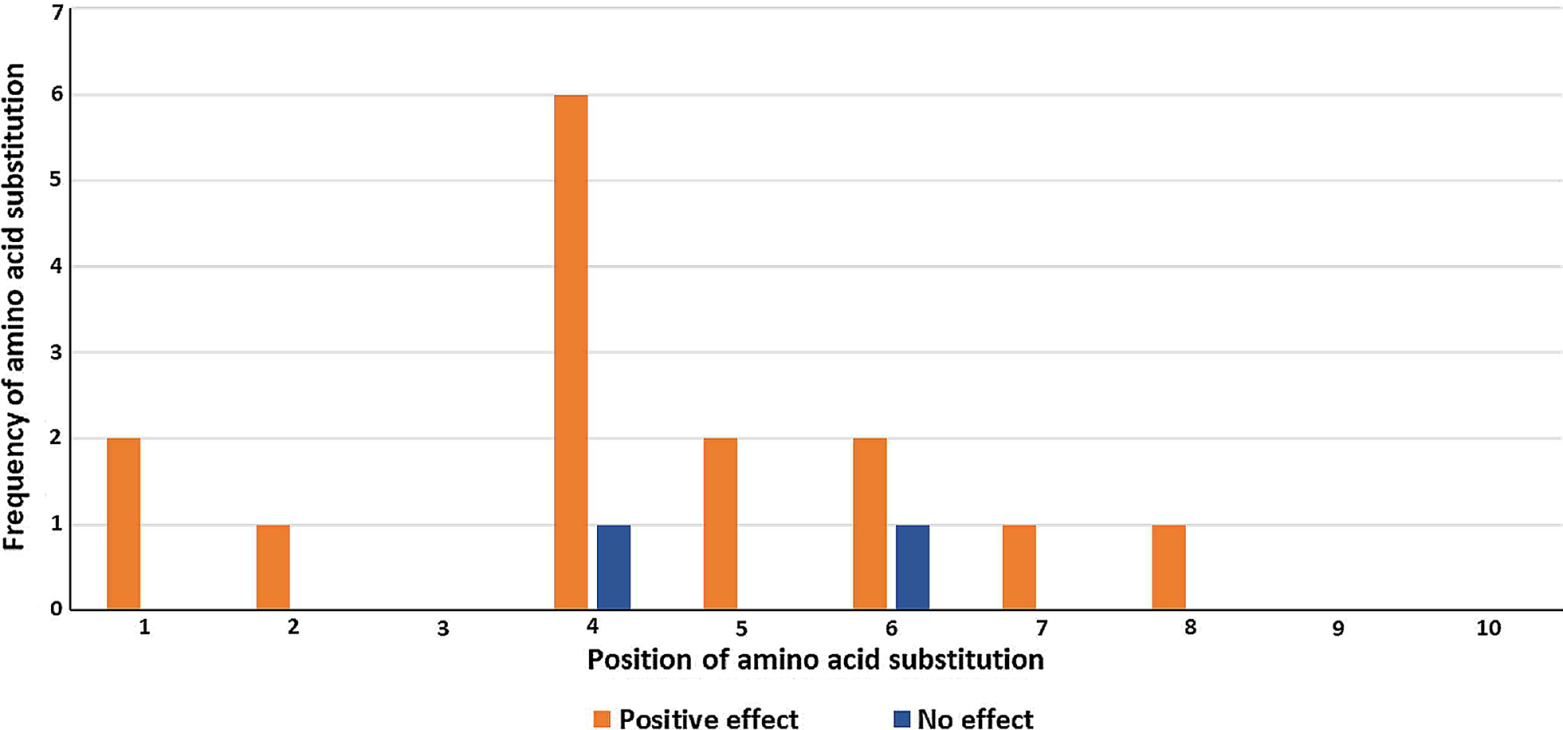
Positions and frequency of amino acid substitutions in peptides with positive responses in all assays. Orange bars represent amino acid substitutions that can be associated with IFN-**γ** response abrogation, and blue bars represent amino acid substitutions that did not result in abrogation of IFN-**γ** response

Positions and frequencies of amino acids within peptides that made positive responses are shown in Fig. 3. A total of 14 amino acid substitutions were associated with positivity of the tested peptides and these occurred at seven different positions within the peptide’s sequences. These were positions 1, 2, 4, 5, 6, 7 and 8. A total of five substitutions occurred at position 4, and this included substitution in the only peptide that tested positive in the low transmission area. Two substitutions occurred at each of positions 1, 5 and 6, and a single substitution occurred at each of positions 2, 7 and 8.

## Discussion

T lymphocyte recognition of sequences within malaria antigens is an essential feature of the adaptive immune response for limiting *Plasmodium* infection in humans. Interferon-gamma (IFN-γ) is a key immune molecule that is secreted by parasite-specific activated T cells and has a liver stage parasite killing effect. Recognition of parasite antigen peptide-MHC complexes by T cells involves molecular interactions between amino acid residues on both the TCR and the parasite peptides, and polymorphisms within either could potentially affect peptide recognition. In this study, we assessed the IFN-γ induction potential of predicted polymorphic *Pf*AMA1 peptides using a standard ELISpot assay and compared responses between individuals living in high and low malaria transmission areas.

Based on the peptides tested in this study, positive response frequency was higher in Obom relative to Legon. This may be a reflection of the fact that Obom is a high malaria transmission community, and subjects may have been exposed to multiple strains circulating in the community. The observation correlated with the expectation that high transmission most likely drives increased parasite diversity [37, 38]. The multiplicity of infection with parasites has generally been reported to be greater in high transmission areas compared with low transmission areas [39, 40]. It is therefore expected that, persons living in high endemic communities would most likely have concomitant infection with diverse parasite strains, and this will result in the activation of several clones of T cells committed to responding to the different parasite strains. These T cell clones can survive for years [41] and upon re-infection, they can be recalled to mount immune attack on the new parasites. However, populations with low endemicity have a lower proportion of mixed-genotype infections, due to a low rate of superinfection, hence limited diverse clones of T cells. It must be noted that our earlier studies have picked up relatively higher numbers of anti-*Pf*AMA1 responses with subjects recruited from the Legon area, but these studies have mostly tested *Pf*AMA1 peptide pools that span the entire *Pf*AMA1 sequence [32, 42, 43], while the current study tested a limited number of 15 single 9-10mer HLA-restricted minimal epitopes. Five of the eight (63%) peptides that made positive responses in this study were 3D7 *Pf*AMA1 variants (Table 2), and while this could suggest 3D7-like parasites to be the commonest circulating *P. falciparum* strains in the study communities, this observation could also simply be due to the fact that our selection of peptides for testing was based on epitope predictions for the 3D7 variant peptides.

For both study sites, the number of positive peptide responses increased upon CD8 + enrichment of PBMCs. We have previously reported similar findings with CSP peptides [36] and this could be due a depletion of CD4 + regulatory T cell subsets (T regs) from PBMCs. Classically, T regs are a subset of CD4 + T cells [44] and have been shown to increase in frequency during natural malaria infections [45–47]. The hallmark effect of Tregs are the impairment of T cell proliferation and cytokine production from other T cell subsets following engagement of their antigen-specific TCRs [41, 48, 49]. Hence depletion of these subsets may have led to removal of the stimulation restriction effect on CD8 + T cells, resulting in higher IFN-γ activities in some subjects. It is worth noting that the subjects who made positive responses in assays with CD8 + enriched PBMCs (s3, s5, s7 and s17) did not make significant responses with the unfractionated PBMCs. Conversely, in subjects (s2 and s6) whose unfractionated PBMC were positive to some peptides, these effects were lost upon depletion of the CD4 + fraction of PBMCs. On this basis, it is possible that these positive responses seen were CD4 + T cell-specific although we could not confirm this due to insufficient cell numbers to test the CD4 + enriched PBMC.

Amino acid changes that affected peptide positivity in assays mostly occurred at residue positions 4, 5 and 6 (Fig. 3). Jordan et al. [50] reported that substitutions that suitably change the spatial structure of peptides may enhance the immunogenicity of epitopes and improve the binding of TCRs to MHC ligands. Moreover, Calis et al. [51] have reported a significant impact of amino acid residue, weighted by the position at which it is found within the peptide, on the peptide’s immunogenicity. They found that T cells have preference for aromatic and large residues and position 4 to 6 were shown to be most important especially in MHC class I antigen presentation to CD8 + T cells. In this study, substitutions within the peptides that made positive responses mostly occurred within these positions.

In contrast, all peptides within two allelic sets elicited positive responses (Figs. 1 and 2), suggesting that the substitutions within these peptides, which occurred in positions 4 and 6 respectively (Fig. 3), were not enough to abrogate T cell response induction. Thus there was no clear relationship between amino acid residue substitutions within the peptide sequences and their effect on peptide positivity in assays.

This study was limited in two ways. First, very few peptides were tested. Using a greater number of predicted minimal epitopes could have increased our chances of identifying additional immunogenic peptides whose reactivities may be altered by the changes in variant parasite strains. Second, subjects from both sites were not HLA-typed hence the peptides tested were not selected on the basis of their predicted recognition of subjects’ HLA types. Selection of peptides based on study subject HLA types may have increased the positivity rate of the tested peptides [13]. These findings are however relevant for the concept of constituting several T cell epitopes that are identified to be presented by specific HLA alleles into a multi-epitope vaccine for broad population use or as biomarkers for protective T cell immunity. Promiscuity in HLA recognition and binding by immunodominant peptides [52, 53] would contribute to the understanding of how this could work and make it possible for such potential multi-epitope vaccines to work in persons with HLA types that are not those upon which the vaccine epitopes were identified.

## Conclusion

This study has provided some evidence that polymorphisms existing in peptides from malaria vaccine candidate antigens, like *Pf*AMA1, can affect peptide recognition by T cells and hence the immune response that will be elicited. Furthermore, high endemicity which is expected to drive greater parasite diversity, may result in a broadening of the repertoire of T cells that can recognize specific peptides beyond what can be achieved with a whole parasite vaccine containing a single parasite strain. We can also infer the possibility of broad HLA recognition of peptides originally predicted to be presented by specific HLA alleles, and this could further strengthen the multi-epitope vaccine design concept. These findings will further our understanding of cellular immune mechanisms that govern anti-*Plasmodium* T cell responses and help direct the selection of peptides for inclusion in multi-epitope vaccines that can offer cross-strain protection as well as biomarkers for assessing protective T cell immunity.

## Supplementary Information

**Additional file 1.** Complete data for subjects whose PBMCs showed at least one positive peptide response. Peptide and positive control (con A) sfc/m data are after subtraction of the corresponding medium only sfc/m for that subject.

**Additional file 2.** Representative sample of CD8 + T cells enrichment confirmed by flow cytometry. Figure A shows the population of non-CD8 + cells (CD3 + CD8-) and CD8 + T cells (CD3 + CD8 +) before negative selection. Figure B shows the population of non-CD8 + cells (CD3 + CD8-) and CD8 + T cells (CD3 + CD8 +) after negative selection.

## Data Availability

All data generated or analysed during this study are included in this published article.

## Abbreviations

*Pf*AMA1: *P. falciparum* apical membrane antigen 1
CSP: Circumsporozoite protein
ELISpot: Enzyme-linked immunospot assay
HLA: Human leukocyte antigen
IFN-γ: Interferon-gamma
MHC: Major histocompatibility complex
NMIMR: Noguchi Memorial Institute for Medical Research
PBMC: Peripheral blood mononuclear cell
RDT: Rapid diagnostic test
Sfc/m: Spot forming Cells per million
TCR: T cell receptor
WHO: World Health Organization
