# Supplementary material for: Comparison of the impact of allelic polymorphisms in PfAMA1 on the induction of T Cell responses in high and low malaria endemic communities in Ghana
Source: Malar J. 2021 Sep 10;20:367. doi: 10.1186/s12936-021-03900-1 (PMC8431259; doi:10.1186/s12936-021-03900-1)
Supplement: Supplementary file 1 — Additional file 1. Complete data for subjects whose PBMCs showed at least one positive peptide response. Peptide and positive control (con A) sfc/m data are after subtraction of the corresponding medium only sfc/m for that subject. [file 12936_2021_3900_MOESM1_ESM.docx]

**Additional file 1. Subject-specific whole PBMC and CD8+ enriched PBMC responses to all stimulants**

| Study subject | Peptide | Sequence | Strain | Sfc/million PBMC | |
| --- | --- | --- | --- | --- | --- |
|  |  |  |  | Unfracti-onated PBMC | CD8+ enriched PBMC |
| s3 | e1 | AKD**KL**F**E**NY | FVO | 143 | 41 |
|  | e2 | AKD**K**SFQNY | 7G8 | 159 | 13 |
|  | e3 | AKDISFQNY | 3D7 | 171 | 16 |
|  | e22 | DVY**H**PINEHR | 7G8, FVO | 183 | 13 |
|  | e23 | DVYRPINEHR | 3D7 | 255 | 19 |
|  | e24 | EHREH**S**KEY | 7G8 | 228 | 20 |
|  | e25 | EHREHPKEY | 3D7, FVO | 261 | 40 |
|  | e46 | **D**FYK**N**N**E**YVK | 7G8, FVO | 209 | 35 |
|  | e48 | HFYKDNKYVK | 3D7 | 148 | **55+** |
|  | e60 | **KL**F**E**NYTYL | FVO | 169 | 35 |
|  | e61 | **K**SFQNYTYL | 7G8 | 173 | 5 |
|  | e62 | ISFQNYTYL | 3D7 | 234 | 8 |
|  | e87 | MTL**NG**MR**D**FY | FVO | 255 | 10 |
|  | e88 | MTLD**H**MR**D**FY | 7G8 | 234 | 15 |
|  | e89 | MTLDEMRHFY | 3D7 | 305 | 18 |
|  | NC | Medium | - | **276** | **26** |
|  | PC | Con A | - | **719** | **151** |

| Study subject | Peptide | Sequence | Strain | Sfc/million PBMC | |
| --- | --- | --- | --- | --- | --- |
|  |  |  |  | Unfracti-onated PBMC | CD8+ enriched PBMC |
| s2 | e1 | AKD**KL**F**E**NY | FVO | 74 | 61 |
|  | e2 | AKD**K**SFQNY | 7G8 | 133 | 56 |
|  | e3 | AKDISFQNY | 3D7 | 85 | 60 |
|  | e22 | DVY**H**PINEHR | 7G8, FVO | 93 | 64 |
|  | e23 | DVYRPINEHR | 3D7 | 68 | 56 |
|  | e24 | EHREH**S**KEY | 7G8 | 58 | 48 |
|  | e25 | EHREHPKEY | 3D7, FVO | 1**80+** | 70 |
|  | e46 | **D**FYK**N**N**E**YVK | 7G8, FVO | 156 | 43 |
|  | e48 | HFYKDNKYVK | 3D7 | 163 | 61 |
|  | e60 | **KL**F**E**NYTYL | FVO | 166 | 38 |
|  | e61 | **K**SFQNYTYL | 7G8 | 91 | 55 |
|  | e62 | ISFQNYTYL | 3D7 | 69 | 49 |
|  | e87 | MTL**NG**MR**D**FY | FVO | 74 | 44 |
|  | e88 | MTLD**H**MR**D**FY | 7G8 | 36 | 64 |
|  | e89 | MTLDEMRHFY | 3D7 | 28 | 55 |
|  | NC | Medium | - | **88** | **55** |
|  | PC | Con A | - | **284** | **234** |

| Study subject | Peptide | Sequence | Strain | Sfc/million PBMC | |
| --- | --- | --- | --- | --- | --- |
|  |  |  |  | Unfracti-onated PBMC | CD8+ enriched PBMC |
| s5 | e1 | AKD**KL**F**E**NY | FVO | 96 | 28 |
|  | e2 | AKD**K**SFQNY | 7G8 | 86 | 40 |
|  | e3 | AKDISFQNY | 3D7 | 116 | 40 |
|  | e22 | DVY**H**PINEHR | 7G8, FVO | 73 | 26 |
|  | e23 | DVYRPINEHR | 3D7 | 114 | **60+** |
|  | e24 | EHREH**S**KEY | 7G8 | 113 | 44 |
|  | e25 | EHREHPKEY | 3D7, FVO | 101 | **56+** |
|  | e46 | **D**FYK**N**N**E**YVK | 7G8, FVO | 79 | 25 |
|  | e48 | HFYKDNKYVK | 3D7 | 88 | 19 |
|  | e60 | **KL**F**E**NYTYL | FVO | 83 | 20 |
|  | e61 | **K**SFQNYTYL | 7G8 | 80 | 20 |
|  | e62 | ISFQNYTYL | 3D7 | 103 | 40 |
|  | e87 | MTL**NG**MR**D**FY | FVO | 111 | 45 |
|  | e88 | MTLD**H**MR**D**FY | 7G8 | 93 | 29 |
|  | e89 | MTLDEMRHFY | 3D7 | 78 | **61+** |
|  | NC | Medium | - | **73** | **26** |
|  | PC | Con A | - | **321** | **199** |

| Study subject | Peptide | Sequence | Strain | Sfc/million PBMC | |
| --- | --- | --- | --- | --- | --- |
|  |  |  |  | Unfracti-onated PBMC | CD8+ enriched PBMC |
| s6 | e1 | AKD**KL**F**E**NY | FVO | 36 | 205 |
|  | e2 | AKD**K**SFQNY | 7G8 | 35 | 184 |
|  | e3 | AKDISFQNY | 3D7 | **89+** | 181 |
|  | e22 | DVY**H**PINEHR | 7G8, FVO | **101+** | 178 |
|  | e23 | DVYRPINEHR | 3D7 | 36 | 194 |
|  | e24 | EHREH**S**KEY | 7G8 | **73+** | 174 |
|  | e25 | EHREHPKEY | 3D7, FVO | **48+** | 181 |
|  | e46 | **D**FYK**N**N**E**YVK | 7G8, FVO | 28 | 213 |
|  | e48 | HFYKDNKYVK | 3D7 | 25 | 181 |
|  | e60 | **KL**F**E**NYTYL | FVO | 9 | 209 |
|  | e61 | **K**SFQNYTYL | 7G8 | 6 | 165 |
|  | e62 | ISFQNYTYL | 3D7 | 13 | 154 |
|  | e87 | MTL**NG**MR**D**FY | FVO | 11 | 183 |
|  | e88 | MTLD**H**MR**D**FY | 7G8 | 21 | 188 |
|  | e89 | MTLDEMRHFY | 3D7 | 16 | 199 |
|  | NC | Medium | - | **20** | **181** |
|  | PC | Con A | - | **804** | **310** |

| Study subject | Peptide | Sequence | Strain | Sfc/million PBMC | |
| --- | --- | --- | --- | --- | --- |
|  |  |  |  | Unfracti-onated PBMC | CD8+ enriched PBMC |
| s17 | e1 | AKD**KL**F**E**NY | FVO | 4 | 48 |
|  | e2 | AKD**K**SFQNY | 7G8 | 8 | 43 |
|  | e3 | AKDISFQNY | 3D7 | 10 | 39 |
|  | e22 | DVY**H**PINEHR | 7G8, FVO | 4 | 45 |
|  | e23 | DVYRPINEHR | 3D7 | 5 | **56+** |
|  | e24 | EHREH**S**KEY | 7G8 | 9 | 36 |
|  | e25 | EHREHPKEY | 3D7, FVO | 10 | 46 |
|  | e46 | **D**FYK**N**N**E**YVK | 7G8, FVO | 8 | 41 |
|  | e48 | HFYKDNKYVK | 3D7 | 10 | 19 |
|  | e60 | **KL**F**E**NYTYL | FVO | 4 | 28 |
|  | e61 | **K**SFQNYTYL | 7G8 | 1 | 44 |
|  | e62 | ISFQNYTYL | 3D7 | 5 | 41 |
|  | e87 | MTL**NG**MR**D**FY | FVO | 1 | 48 |
|  | e88 | MTLD**H**MR**D**FY | 7G8 | 1 | 44 |
|  | e89 | MTLDEMRHFY | 3D7 | 1 | 50 |
|  | NC | Medium | - | **4** | **25** |
|  | PC | Con A | - | **170** | **225** |

| Study subject | Peptide | Sequence | Strain | Sfc/million PBMC | |
| --- | --- | --- | --- | --- | --- |
|  |  |  |  | Unfracti-onated PBMC | CD8+ enriched PBMC |
| s7 | e1 | AKD**KL**F**E**NY | FVO | 43 | 50 |
|  | e2 | AKD**K**SFQNY | 7G8 | 61 | **58+** |
|  | e3 | AKDISFQNY | 3D7 | 54 | 50 |
|  | e22 | DVY**H**PINEHR | 7G8, FVO | 50 | **58+** |
|  | e23 | DVYRPINEHR | 3D7 | 55 | **54+** |
|  | e24 | EHREH**S**KEY | 7G8 | 51 | 38 |
|  | e25 | EHREHPKEY | 3D7, FVO | 43 | 34 |
|  | e46 | **D**FYK**N**N**E**YVK | 7G8, FVO | 61 | 43 |
|  | e48 | HFYKDNKYVK | 3D7 | 34 | 31 |
|  | e60 | **KL**F**E**NYTYL | FVO | 45 | 29 |
|  | e61 | **K**SFQNYTYL | 7G8 | 49 | 25 |
|  | e62 | ISFQNYTYL | 3D7 | 65 | 40 |
|  | e87 | MTL**NG**MR**D**FY | FVO | 76 | 25 |
|  | e88 | MTLD**H**MR**D**FY | 7G8 | 58 | 38 |
|  | e89 | MTLDEMRHFY | 3D7 | 79 | 36 |
|  | NC | Medium | - | **63** | **26** |
|  | PC | Con A | - | **296** | **293** |
