# Supplementary figures and images for: Comparison of the impact of allelic polymorphisms in PfAMA1 on the induction of T Cell responses in high and low malaria endemic communities in Ghana
Source: Malar J. 2021 Sep 10;20:367. doi: 10.1186/s12936-021-03900-1 (PMC8431259; doi:10.1186/s12936-021-03900-1)

**A**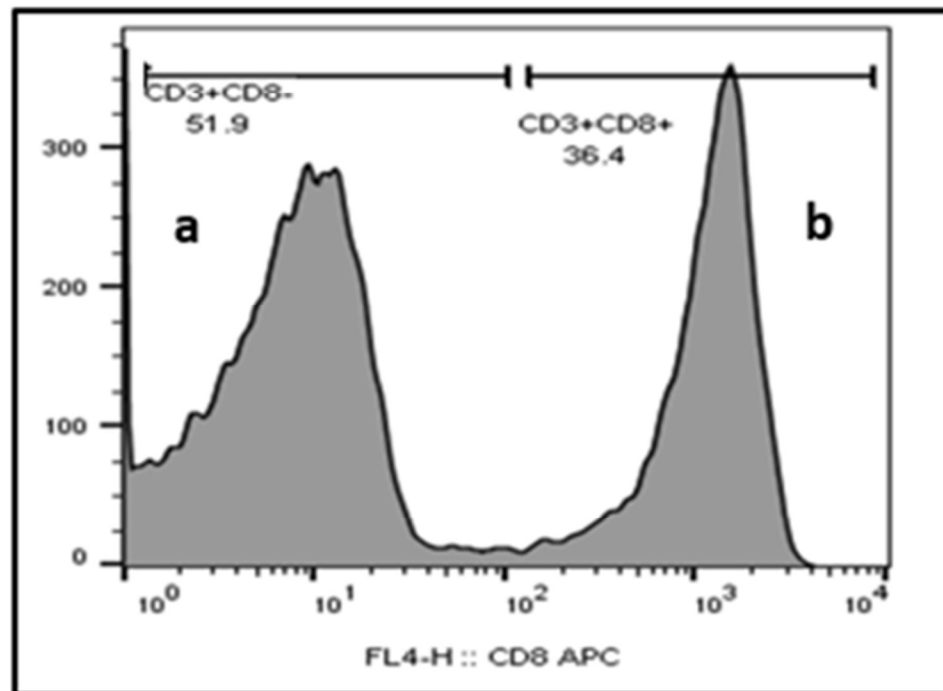**B**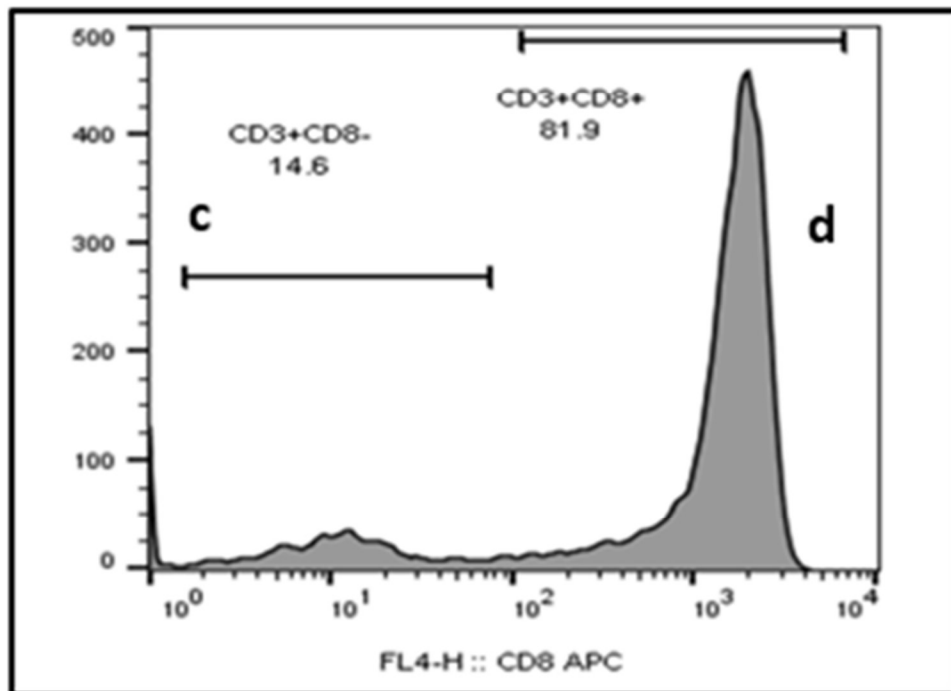

Supplement: Supplementary file 2 — Additional file 2. Representative sample of CD8 + T cells enrichment confirmed by flow cytometry. Figure A shows the population of non-CD8 + cells (CD3 + CD8-) and CD8 + T cells (CD3 + CD8 +) before negative selection. Figure B shows the population of non-CD8 + cells (CD3 + CD8-) and CD8 + T cells (CD3 + CD8 +) after negative selection. [file 12936_2021_3900_MOESM2_ESM.pdf]
